# Prognostic factors and epidemiology of adult open globe injuries from Western Sydney: a twelve-year review

**DOI:** 10.1186/s12886-021-01929-z

**Published:** 2021-04-10

**Authors:** Haochi Ho, Jane Foo, Yi-Chiao Li, Samantha Bobba, Christopher Go, Jaya Chandra, Adrian T. Fung

**Affiliations:** 1 Department of Ophthalmology, Westmead Hospital, Corner of Hawkesbury and Darcy Roads, Westmead, Sydney, NSW 2145 Australia; 2 Sarawak General Hospital, Kuching, Sarawak Malaysia; 3 Hospital Raja Permaisuri Bainun, Ipoh, Perak Malaysia; 4 Westmead and Central Clinical Schools, Discipline of Ophthalmology and Eye Health, The University of Sydney, Sydney, Australia; 5 Faculty of Medicine, Health and Human Sciences, Macquarie University Hospital, Sydney, Australia

**Keywords:** Open globe injury, Globe rupture, Retinal detachment

## Abstract

**Background:**

To identify prognostic factors determining final visual outcome following open globe injuries.

**Methods:**

Retrospective case series of patients presenting to Westmead Hospital, Sydney, Australia with open globe injuries from 1st January 2005 to 31st December 2017. Data collected included demographic information, ocular injury details, management and initial and final visual acuities.

**Results:**

A total of 104 cases were identified. Predictors of poor final visual outcomes included poor presenting visual acuity (*p* < 0.001), globe rupture (p < 0.001), retinal detachment (*p* < 0.001), Zone III wounds (p < 0.001), hyphema (*p* = 0.003), lens expulsion (p = 0.003) and vitreous hemorrhage (*p* < 0.001). Multivariate analysis demonstrated presenting visual acuity (p < 0.001), globe rupture (*p* = 0.013) and retinal detachment (*p* = 0.011) as being statistically significant for predicting poor visual outcomes. The presence of lid laceration (*p* = 0.197) and uveal prolapse (*p* = 0.667) were not significantly associated with the final visual acuity.

**Conclusions:**

Poor presenting visual acuity, globe rupture and retinal detachment are the most important prognostic factors determining final visual acuity following open globe injury.

## Background

Open globe injury (OGI) is defined as a full-thickness injury to the eyewall. Blunt trauma may cause globe rupture, whereas sharp trauma may be associated with penetrating or perforating injuries with or without an intraocular foreign body (IOFB). The incidence of OGI in Australia has been reported to be 3.7/100000 per annum [1, 2], similar to the United States at 3.49/100000 per annum [3]. Our understanding of OGIs has increased tremendously over the past decade thanks to the standardisation of terminology [4, 5] and prognostic parameters based on the Ocular Trauma Classification [6]. To our knowledge, there is no report on OGIs from Western Sydney. The closest we can find was a study on open globe injuries from 2010 to 2015 from Sydney Eye Hospital [7].

The purpose of this study was to identify prognostic factors for final visual outcome following OGIs presenting to a tertiary teaching hospital in Western Sydney, Australia.

## Methods

A retrospective review of all patients with open globe injuries presenting to Westmead Hospital, Western Sydney Local Health District (WSLHD), Sydney, New South Wales, Australia from 1st January 2005 to 31st December 2017 was performed. Ethics approved via the WSLHD Human Ethics Research Committee. Medical records were searched for the following diagnostic codes: S05.4 “Penetrating wound of orbit with or without foreign body”, S05.5 “Penetrating wound of eyeball with foreign body” or S05.6 “Penetrating wound of eyeball without foreign body” to identify relevant cases. Clinical notes, operative reports and any imaging performed (B-scans, X-rays, CT scans) were reviewed.

Data collected included: patient demographics, history of prior ocular trauma, cause of injury (hammer/ chisel, assault, fall, motor vehicle accident (MVA) or explosion/ blast), setting of injury (home or work-related), association with alcohol or drug use, laterality and presenting and final visual acuity (VA). Best-corrected VA was recorded from refracted vision, or pinhole vision if refraction was not performed. Snellen visual acuity was converted to LogMAR values. The types of injury were classified according to the Birmingham Eye Trauma Terminology (BETT) into globe rupture, penetrating injury, perforating injury and/or intraocular foreign bodies [5]. Wound location was classified into Zone I (full-thickness wound involving the cornea only), Zone II (when the wound involves sclera not more posteriorly than 5 mm from the corneoscleral limbus) or Zone III (when the wound is posterior to Zone II) [6]. The presence or absence of a relative afferent pupillary defect (RAPD), retinal detachment, hyphema, lens expulsion, vitreous hemorrhage, eyelid laceration or uveal prolapse at presentation were recorded. The ocular management and development of complications were also noted.

Statistical analysis was performed using SPSS Statistics v23 (IBM). Simple linear regression was used to correlate the presenting VA to the final VA. Independent T-test was used to analyse each parameter. Generalised linear modelling was used for multivariant analysis to determine the main outcome predictors.

## Results

A total of 104 eyes in 104 patients were identified. The mean age was 43 years (19 to 89, ± 17 years SD). The median follow-up period was 17 months (range, 7 to 118 months).

The most common cause of injury was from a hammer or chisel (33.7%) followed by “other” (29.8%), assault (14.4%), falls (10.6%), motor vehicle accidents (6.7%) and explosions/blasts (4.8%). One-fifth of cases were work-related (20%). Six (6%) cases were related to substance abuse (4 cases of alcohol-related assault, 1 alcohol-related fall and 1 drug-related assault). Eight patients (7.7%) had previous ocular trauma (Table 1). Three patients had previous trauma to the same eye. Five patients had previous trauma to the other eye. One patient had a dehiscence of penetrating keratoplasty from jumping into a pool of water.

**Table 1:** Baseline Data

Demographic data	n (%)
Sex	
Male	88 (85%)
Female	16 (15%)
Laterality	
Right	55 (53%)
Left	49 (47%)
Work-related	
Yes	21 (20%)
No	83 (80%)
Previous ocular trauma	
Yes	8 (8%)
No	96 (92%)
Substances abuse-related	
Yes	6 (6%)
No	98 (94%)

**Fig. 1:**
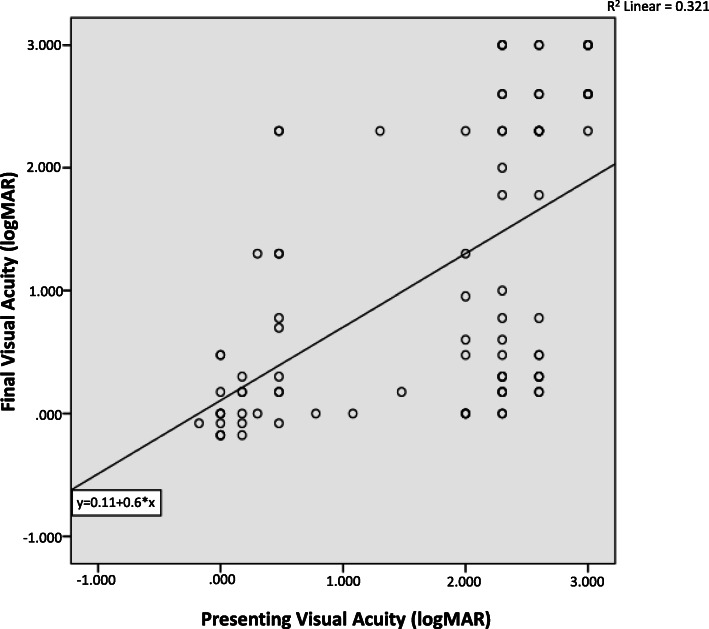
Graph of Presenting Versus Final Visual Acuity

Presenting VA was found to be a very strong predictor of final VA as shown in the linear regression model (*p* < 0.001). The patients stratified into two main groups of presenting VAs- equal or better than LogMAR 0.60 and LogMAR 2.00 or worse. Patients presenting with VA ≤ LogMAR 0.60 usually had good final VA. In contrast, the group with initial VA ≥ LogMAR 2.00 was quite variable, some achieved excellent results while some did very poorly (Fig. 1).

The type of injury was strongly correlated with final VA (*p* < 0.001). Penetrating eye injuries (LogMAR 0.80 ± 1.01) and IOFBs (LogMAR 0.84 ± 1.04) did very well, whereas globe ruptures did very poorly (LogMAR 2.28 ± 0.94). The presence of a retinal detachment was strongly correlated with final VA (*p* < 0.001). More posterior wounds were associated with worse final VAs (p < 0.001) with the mean final VAs in Zones I, II and III being LogMAR 0.86 ± 1.01, LogMAR 1.01 ± 1.16 and LogMAR 2.25 ± 1.01 respectively. Other statistically significant poor visual prognostic parameters were hyphema (*p* = 0.003), lens expulsion (p = 0.003) and vitreous hemorrhage (*p* < 0.001). Lid laceration (*p* = 0.197) and uveal tissue prolapse (*p* = 0.667) had no significant effect on the final visual outcome (Table 2). All hyphema subjects included in this study had macroscopic hyphema. All retinal detachment patients were macula-off.

**Table 2:** Clinical Factors Affecting Final Visual Acuity (LogMAR)

Variables	n (%)	Final visual acuity (LogMAR)		***P***-value
		Mean	Standard Deviation	
Types of injury				
Globe rupture	24 (23%)	2.28	0.94	< 0.001
IOFB	17 (16%)	0.84	1.04	
Penetrating eye injury	63 (61%)	0.80	1.01	
Retinal detachment				
Yes	17 (16%)	2.34	0.73	< 0.001
No	87 (84%)	0.92	1.09	
Wound Location				
Zone I	48 (46%)	0.86	1.01	< 0.001
Zone II	39 (38%)	1.01	1.16	
Zone III	17 (16%)	2.25	1.01	
Hyphema				
Yes	45 (43%)	1.53	1.22	0.003
No	59 (57%)	0.84	1.04	
Lens expulsion				
Yes	12 (12%)	2.08	1.08	0.003
No	92 (88%)	1.02	1.13	
Vitreous hemorrhage				
Yes	26 (25%)	1.82	1.13	< 0.001
No	78 (75%)	0.92	1.09	
Eyelid laceration				
Yes	16 (15%)	1.49	1.32	0.197
No	88 (85%)	1.08	1.14	
Uveal prolapse				
Yes	43 (41%)	1.20	1.20	0.667
No	61 (59%)	1.10	1.15	

A multivariate analysis using generalised linear model (GLM) of factors affecting final VA was carried out with SPSS Statistics v23 (Table 3). The assumptions of homogeneity of variances and normal distribution of residuals were checked and found to be valid. Three predictors of final VA were found to be strongly significant: presenting VA, mechanism of injury and retinal detachment. The other predictors were correlated with these three variables and could be omitted from the model without adversely affecting the goodness of fit. No interactions were found to be of statistical significance. The final GLM model with the three factors had an F-statistic of 14.736 (*p* < 0.001) and R^2^ of 0.461, suggesting a high level of relationship between the factors and final VA, and adequate goodness of fit. The parameter estimated for presenting VA predicting final VA was 0.41, which means each 1 logMAR of reduction in presenting VA results in 0.41 reduction of final VA (95% CI 0.22–0.60). Globe rupture had the worst prognosis of all injury mechanisms. Compared with globe ruptures, the final VA for penetrating eye injuries were better by a mean of 0.87 logMAR (95% C.I. 0.36–1.38) and IOFB were better by 0.72 logMAR (95% C.I. 0.08–1.36). The presence of retinal detachment worsened final VA by 0.73 logMAR (95% C.I. 0.17 to 1.29).

**Table 3:** Parameter Estimates From Generalised Linear Modelling Showing the Size of Effect of Significant Factors Affecting Final Visual Acuity

Parameter	Estimate	Standard Error	P-value	95% confidence interval
Presenting Visual Acuity	0.41	0.10	< 0.001	0.216 to 0.597
Injury Type	Globe rupture = 0			
	Penetrating = −0.87	0.26	0.001	−1.378 to − 0.358
	IOFB = − 0.72	0.32	0.028	−1.364 to − 0.078
	Not specified = − 0.53	0.92	0.563	−2.355 to 1.290
Retinal Detachment	Absent = 0 Present = 0.73	0.28	0.011	0.171 to 1.285

Of the 104 eyes, 97 (93%) eyes needed primary repair, with 7 (7%) presenting with self-sealing wounds. The mean duration from trauma to wound closure was 0.84 days (SD 1.95 days). Seventeen (16.3%) eyes received intravitreal antibiotics and 19 (18.3%) received intracameral antibiotics intraoperatively during primary surgery. While 93 (89.4%) patients received systemic antibiotics on presentation.

Thirty patients (28.8%) patients underwent pars plana vitrectomy (PPV). Retinal detachment (RD) was the most common indication for PPV with 15 cases. Other indications for PPV were: 5 IOFB, 4 dislocated lens, 3 vitreous hemorrhage, 2 endophthalmitis and 1 epiretinal membrane. 4 out of 5 (80%) IOFB, 1 out of 2 (50%) dislocated lens, 1 out of 2 (50%) endophthalmitis, 5 out of 15 (33.3%) of RD had PPV within 2 weeks post-trauma. All PPVs were performed using 23G or 25G vitrector cutters.

Complications included 3 eyes with persistent wound leak after their primary repair. All 3 had re-suturing and 1 needed additional glue. There were 5 eyes that developed post-traumatic endophthalmitis. Another patient developed post-operative endophthalmitis following subsequent cataract surgery 5 months after the globe injury. Two cases underwent subsequent evisceration for painful blind eyes. There was no case of sympathetic ophthalmia.

## Discussion

The follow-up period for final visual acuity varied greatly because some patients had no procedure (self-sealing wounds) while some had multiple corrective surgeries over a long period (up to 6 surgeries). BCVA was taken at least 6 months after the last surgery or presentation if no surgery was performed.

The mean age for our study patients was 43-years-old (range, 18 to 89 years old), which falls within the range of means reported by other Australian studies (30.4 to 44.8 years old) [1, 2, 8]. Westmead Hospital is an adult hospital and only accepts patients 14 years 9 months and older, so the demographic will be skewed towards older ages. The male preponderance (84.6%) in our cohort is slightly more than reported in other Australian studies (77.4–83.0%) [1, 2, 8] and much higher than reported in Japan (66.1%) [9]. In our study, 21.3% of OGI was work-related, similar to other Australian studies (18.6–38.8%) [1, 2, 8] but less than that reported in a Japanese study (45.8%) [9]. These variations could be due to the differences in culture and lifestyle. Besides that, the definition of work-related injury is loose. For instance, injuries sustained whilst working at home or not covered by workers’ compensation may not have been counted.

Six cases (6%) of substance-related OGI were reported in our study (4 alcohol and 1 drug-related assaults with 1 alcohol-related fall). Thirty percent of the OGI caused by assaults were substance-related. This is half of what has been reported in another Australian study, where 76.2% of assaults were alcohol-related [1]. In that study, the majority of the alcohol related assaults occurred in Aboriginals and Torres Strait Islanders, a demographic that is not frequently seen at Westmead Hospital [1].

Multivariate analysis identified three main predictors of final VA: presenting VA, type of injury and presence of a retinal detachment. Although other factors such as zones of wound location, hyphema, lens expulsion and vitreous hemorrhage were also associated with final VA outcomes, they were all correlated with each other. For example, a ruptured globe is likely to have hyphema, vitreous hemorrhage and lens expulsion as well.

The Ocular Trauma Classification Group based its classification on four variables: initial VA, mechanism of injury, zone of wound location and presence of a RAPD as accurate predictors of final VA [6]. This is broadly in agreement with our study except we didn’t analyse RAPD due to incomplete data (2 positive RAPD, 17 negative RAPD and 85 undocumented). However, we found another parameter, retinal detachment to be a strong predictive factor. It is possible that in the absence of a documented RAPD, retinal detachment may be used as a replacement for outcome prediction since a RAPD is often present when there is a large retinal detachment.

Initial VA is well established as one of the most important parameters determining final visual outcomes [2, 6, 8, 9]. Good vision reflects mild ocular damage, whereas poor vision reflects more extensive destruction which could result in injuries such as retinal detachment and vitreous hemorrhage [6]. In line with previous studies, our results show that globe rupture carries a poor visual prognosis [2, 6, 8]. Globe rupture which resulted from blunt injury causes more diffuse damage compared to sharp injuries such as penetrating eye injuries and IOFBs which cause local damage [6]. In agreement with previous studies [6, 9], Zone III wounds were associated with a poorer visual prognosis in our study. These wounds involve posterior structures such as the retina and optic nerve which heal poorly and lack regenerating ability [6].

Retinal detachment, hyphema, lens expulsion and vitreous hemorrhage have been reported to be associated with poor visual outcomes [2, 6, 8, 9]. This usually results from severe ocular trauma in association with other ocular tissue damage. In agreement with some [2] but not all [8] studies, uveal prolapse was not shown to be a significant predictor of final visual outcome.

Eyelid laceration has previously been reported to be associated with poor outcomes [10]. Lid laceration and adnexal injuries have been associated with blunt trauma and severe ocular injury with increased likelihood of posterior globe injuries [10]. However, we did not find this association in our study and had more sharp injuries such as penetrating eye injury (*n* = 8) and IOFB (*n* = 1) than globe ruptures (*n* = 7) associated with lid laceration.

The numbers of vitrectomised eyes are not large enough for meaningful analysis of correlation between timing of surgery and visual acuity. However, we note that the indication for early vitrectomy (within 2 weeks post-trauma) was highest for IOFB (80%), followed by lens dislocation (50%), endophthalmitis (50%), retinal detachment (33.3%), vitreous hemorrhage (0%) and epiretinal membrane (0%). This likely reflects the urgency required to remove an IOFB, delayed presentations of endophthalmitis, retinal detachment and epiretinal membrane, delayed diagnosis of retinal detachment in a traumatised eye with media opacity and decisions to observe vitreous hemorrhage which may spontaneously resolve.

Our study has limitations including its retrospective nature, poor documentation of RAPD, use of pin-hole instead of refracted BCVA in some patients and poor documentation of the time of injury, meaning that the time of wound closure could only be calculated to days and not hours. Despite this, it is a reasonably large cohort with significant findings of those parameters which were accurately documented.

## Conclusion

Poor presenting visual acuity, globe rupture and retinal detachment are the most important prognostic factors determining final visual acuity following OGI. The presence of retinal detachment should be considered in future classification schemes of OGI, especially when the presence of a RAPD has not been documented. Uveal prolapse and eyelid laceration had no statistically significant effect on visual prognosis.

## Data Availability

The datasets generated and/or analysed during the current study are not publicly available as individual privacy may be compromised but can be made available from the corresponding author on reasonable request.
